# Manual neuronavigation for superior semicircular canal dehiscence surgery

**DOI:** 10.3389/fneur.2023.1105869

**Published:** 2023-03-30

**Authors:** Nasser Altamami, Michel Khoury, Issam Saliba

**Affiliations:** ^1^Division of Otorhinolaryngology and Head & Neck Surgery, University of Montreal, Montreal, QC, Canada; ^2^Otology and Neurotology, University of Montreal Hospital Center (CHUM), Montreal, QC, Canada

**Keywords:** superior canal, dehiscence, SSCD, craniotomy, plugging, resurfacing, neuronavigation

## Abstract

**Background:**

Intraoperative identification of a superior semicircular canal (SSC) dehiscence *via* the middle cranial fossa approach (MCFA) remains a difficult endeavor without a neuronavigation system. To address these challenges, we propose a technique to localize the SSC dehiscence intraoperatively using certain anatomical landmarks.

**Method:**

Three anatomical landmarks should be identified on preoperative radiological images: the distance from the squamous part of the temporal bone to the dehiscent SSC, the lower limit of the craniotomy, and the exact location of the craniotomy in relation to the bony external auditory canal. The use of these landmarks intraoperatively can allow the surgeon to correctly identify the position of the SSC. Two instructional videos explaining this technique are presented.

**Conclusion:**

The proposed manual neuronavigation technique seems to be an accurate, safe, and cost-effective alternative technique for use in SSC dehiscence surgery.

## Introduction

Since it was first described by Minor et al. in 1998, the presence of a pathological third window in the otic capsule has been found to manifest in a certain proportion of patients, producing severely debilitating symptoms that have a clear effect on their quality of life (1). According to the Classification Committee of the Bárány Society for vestibular disorders, a diagnosis of superior semicircular canal dehiscence (SSCD) requires the presence of four criteria related to the symptoms, clinical signs, and diagnostic tests (2). These criteria, based on the society's recommendations (2), are as follows.

1. At least one of the following symptoms consistent with the presence of a “third mobile window” in the inner ear:

i. Bone conduction hyperacusis;
ii. Sound-induced vertigo and/or oscillopsia time-locked to the stimulus;
iii. Pressure-induced vertigo and/or oscillopsia time-locked to the stimulus;
iv. Pulsatile tinnitus.

2. At least one of the following signs or diagnostic tests indicating a “third mobile window”in the inner ear:

i. Nystagmus characteristic of excitation or inhibition of the affected superior Semicircular canal evoked by sound, or by changes in middle ear pressure or intracranial pressure;
ii. Low-frequency negative bone conduction thresholds on pure tone audiometry;
iii. Enhanced Vestibular evoked myogenic potential (VEMP) responses (low cervical VEMP thresholds or high ocular VEMP amplitudes).

3. High-resolution computed tomography (HRCT) imaging of the temporal bone with multiplanar reconstruction demonstrating dehiscence of the superior semicircular canal.
4. Signs and symptoms are not better accounted for by another vestibular disease or disorder (2).

In fact, most patients with SSCD present with tolerable symptoms and do not need any surgical treatment (3, 4). In accordance with the literature, only a minority of patients presenting with severely intolerable symptoms caused by the dehiscence are candidates for surgical plugging of the defect to improve their quality of life (3, 4). While several surgical techniques exist, our quaternary center promotes the middle cranial fossa approach (MCFA), through which the dehiscent part of the canal can be directly visualized, facilitating surgical plugging of the defect (3).

In the literature, studies have demonstrated radiologically that there is an association between SSCD and tegmen tympani dehiscence in 37%−76% of cases (5, 6). This association may lead to intraoperative confusion in identifying the SSC dehiscence rather than a tegmen tympani defect. Thus, a crucial step in this surgical technique is the correct identification of the dehiscent portion of the SSC, which can easily be mistaken for a dehiscent part of the tegmen tympani. Use of a neuronavigation system in the operating theater can facilitate this difficult step, thereby solving the problem (7). However, the accessibility of this technology remains challenging. In addition, the installation time and maintenance costs of neuronavigation systems limits their widespread use. To address these challenges, we propose a technique for intraoperative localization of the SSC using anatomical landmarks.

## Methods

The patient is first subjected to a high-resolution computed tomography (HRCT) scan with a thin slice thickness of 0.6 mm. Next, preoperative analysis of the HRCT scan using the coronal and axial views is conducted to precisely localize the dehiscence. Three landmarks are identified by the neurotologist surgeon immediately before the surgery. Finally, two measurements are noted on the coronal view.

**Figure 1:**
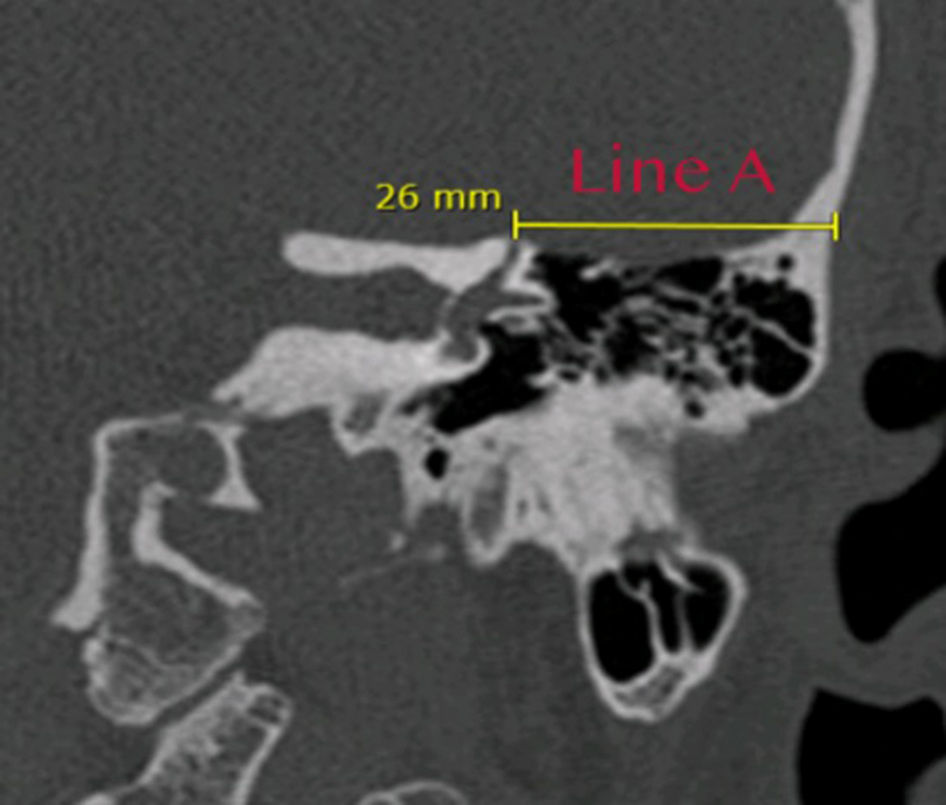
HRCT coronal view illustrating how the first measurement (line A) is taken between the center of the dehiscence medially and the most lateral part of the squamous part of the temporal bone. This line identifies the distance that will be crossed over the superior surface of the temporal bone from the craniotomy laterally to the dehiscence of the superior canal medially, 26 mm in this example.

The first measurement (Figure 1: line A) is taken by drawing a horizontal line on the coronal view HRCT scan between the center of the dehiscent segment of the SSC and the most lateral portion of the squamous part of the temporal bone. In drawing this line, the distance from the craniotomy laterally to the dehiscence of the SSC medially is measured over the temporal bone skull base. This value is important, as it represents the distance that the surgeon will measure to reach the SSC dehiscence medially while dissecting the dura of the temporal lobe.

**Figure 2:**
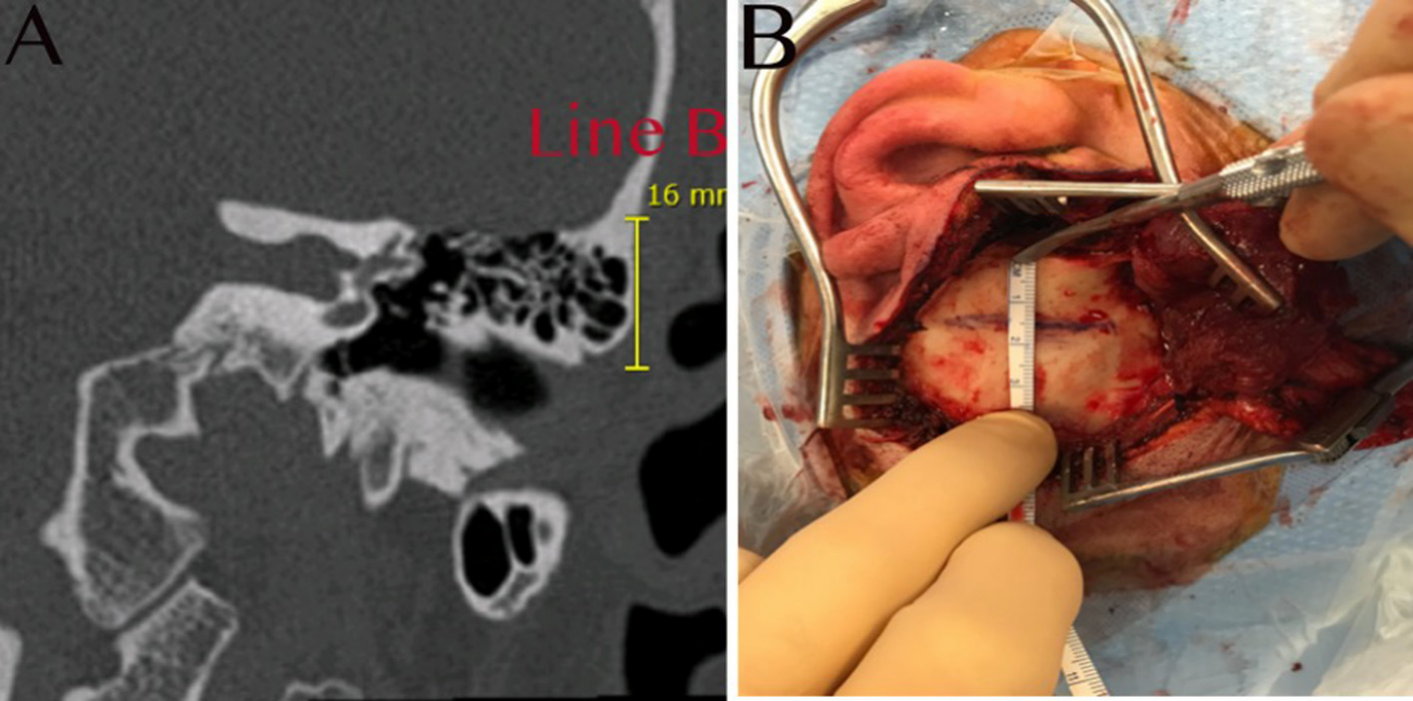
**(A)** HRCT coronal view illustrating how the second measurement (line B) is taken vertically; this will represent the lower limit of the craniotomy to avoid opening the air cells, 16 mm in this example. **(B)** The temporal bone is exposed, and the same measurement is demonstrated, taken from the superior part of the bony external auditory canal to the lower limit of the craniotomy.

The second measurement (Figure 2: line B) determines the height of the lower limit of the craniotomy from the superior bony part of the external auditory canal. This measurement allows the surgeon to avoid opening the pneumatized air cells during the craniotomy. A vertical line is drawn on the same coronal cut used previously, from the tegmen tympani, above the limit of the air cells, to the superior limit of the bony external auditory canal. This distance represents the minimum height for the lower line of the craniotomy to avoid opening the mastoid air cells or the epitympanic cavity and consequently creating a dehiscence of the tegmen tympani or tegmen antri.

**Figure 3:**
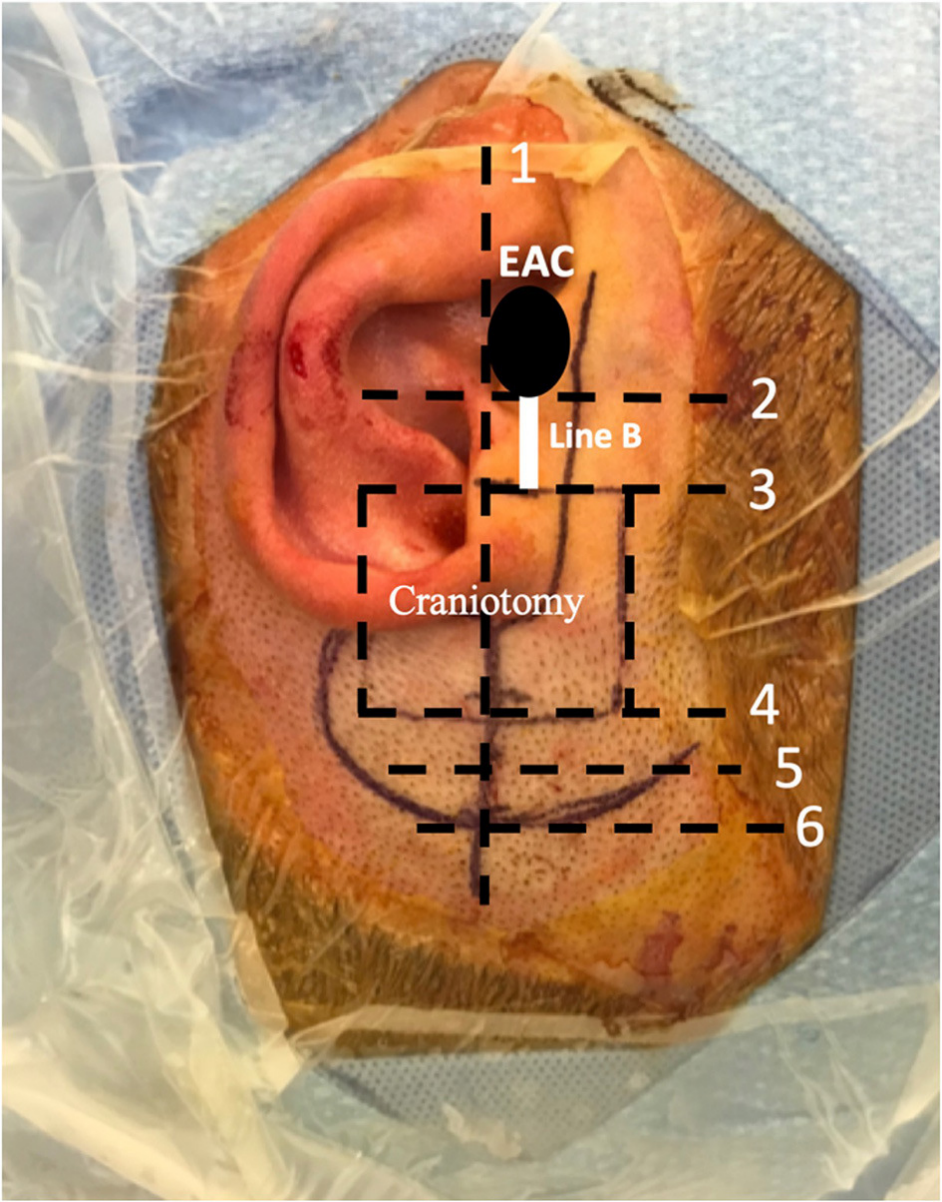
This image illustrates the surgical site and how the incision is made. The surgeon is positioned superiorly near the head of the patient. (1) An imaginary line drawn, in this case posterior to the external auditory canal (EAC), which determines the center of the craniotomy as described in Supplementary Video 1. (2) Superior limit of the bony EAC. (3) Inferior limit of the craniotomy. Line B is the height between lines 2 and 3, 16 mm in this case, as illustrated previously in Figure 2. (4) Superior limit of the craniotomy (2.5 cm × 3 cm or 3 cm × 3 cm). (5) Temporalis muscle flap incision is located 0.5 cm superior to the craniotomy. (6) Superior skin incision limit is located 0.5 cm superior to the temporalis muscle flap incision.

The third measurement point in this manual neuronavigation technique is used to define the anteroposterior location of the craniotomy to position the SSC dehiscence in the middle of the operative field. This third measurement is not a calculated distance *per se*, but it serves to identify the exact position of the craniotomy in order for it to be centered on the SSCD. In the axial view, the surgeon must first identify the dehiscence with the cursor. With the cursor remaining in place, the surgeon must roll through the slices in the inferior direction until they visualize the external auditory canal. At this point, a horizontal line must be drawn from the exact position of the cursor medially to the external auditory canal laterally. This line will pass at the level of the anterior wall, at the level of the posterior wall, or between the two walls, and identifies both the center of the external auditory canal and the center of the craniotomy in the sagittal plane (Supplementary Video 1). Once these three points are radiologically defined, the surgery can begin (Figure 3).

The patient is placed in the supine position. For the patient's security, two straps keep them attached to the table. The head is turned to the opposite side of the dehiscence and the table is then turned toward the opposite side of the operated ear in order to situate the squamous part of the temporal bone in a horizontal position facing the surgeon. The surgeon then sits at the side of the patient's head.

**Figure 4:**
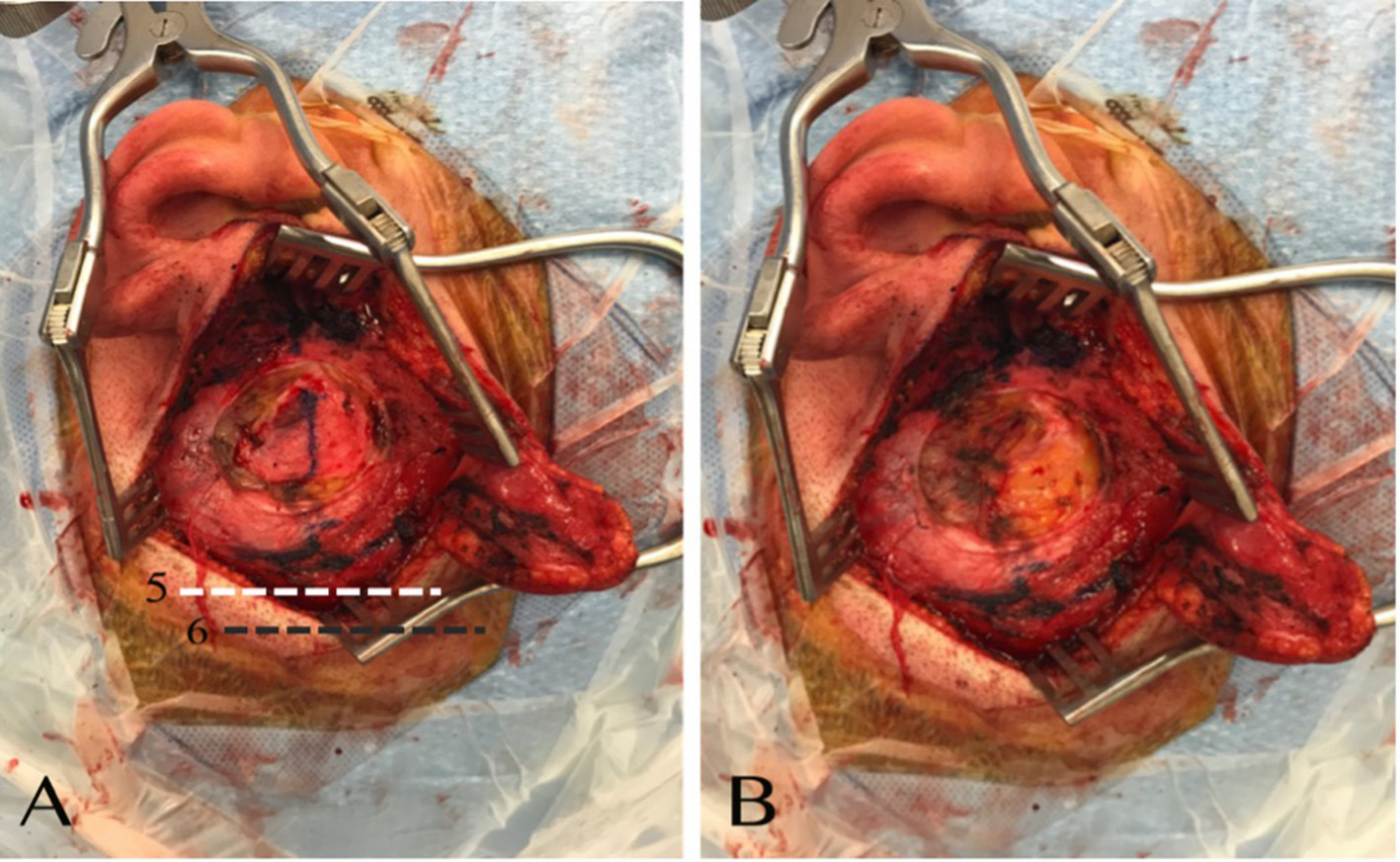
**(A)** The plan over the temporalis fascia is dissected, and the fascia exposed. **(B)** Temporalis fascia is harvested. (5) Demonstration of the level of the temporalis muscle flap incision. (6) Demonstration of the level of the skin incision with respect to the temporalis muscle flap incision.

A question mark-shaped skin incision is made starting anterior to the tragus, passing above the helix posteriorly and turning anteriorly (Figure 3). The dissection is continued above the plane of the temporalis fascia. Next, a 1 × 1 cm temporalis fascia graft is harvested for use as a cover at the end of the procedure; the bone dust is used in plugging the SSCD (Figure 4). Subsequently, careful dissection of the temporalis muscle flap pedicled anteriorly is completed to expose the squamous part of the temporal bone at the site of the craniotomy. In parallel, the superior wall of the external auditory canal should be exposed to measure the height of the inferior limit of the craniotomy (Figure 2B). Next, under a microscope and with a 2-mm cutting burr, the craniotomy (2.5 cm × 3 cm or 3 cm × 3 cm) is made. Bone dust is carefully harvested from the peripheries of the craniotomy. Hemostasis over the dura mater is performed using bipolar cautery.

The dissection of the temporal dura is continued medially toward the superior canal until visualization of the dehiscence. The surgeon should avoid applying any suction on the dehiscent part of the SSC. The middle cranial fossa retractor is used to gently retract the temporal lobe if needed in order to better expose the dehiscence. Once the dehiscence or suspected dehiscence is visualized, the surgeon confirms the dehiscence using a surgical ruler, measuring the distance between the dehiscence and the lateral part of the squamous bone as indicated by line A in the previous drawing (Figure 1). This will precisely confirm the site of the dehiscence. Once the dehiscence is confirmed, the bone dust is used to plug the superior canal and the temporalis is used to cover the bone dust to keep it in place. The craniotomy is closed using four titanium double-Y-shaped plates (Supplementary Video 2). The temporalis muscle and the skin are approximated using absorbable sutures.

We have been using this technique to operate in cases of dehiscence of the superior semicircular canal since 2010. We have operated on 110 patients using this method. A total of 53 other patients were operated on between 2005 and 2010, before we began using this technique.

## Discussion

Repairing a dehiscent SSC remains a technically challenging task, especially without neuronavigation. Given that accessibility, installation time, and maintenance remain the key factors impeding access to this technology, alternative methods are needed to overcome these limitations. To address these challenges, we propose a technique to localize the SSC intraoperatively using anatomical landmarks.

The middle cranial fossa approach is the preferred surgical technique in our center for repair of dehiscence of the SSC, given the optimal surgical exposure that it provides. Specifically, this surgical approach allows direct visualization of the dehiscent area of the canal and permits direct plugging, an option that is not possible when the transmastoid approach (TMA) is used. Through MCFA plugging, there may be a lower risk of sensorineural hearing loss to the SSC and/or vestibular function impairment in the other canals (3, 8). In addition, a recently published study by Renteria et al. demonstrated residual SSC function 3 months postoperatively in 55.3% of patients operated on for SSC dehiscence through the MCFA (9). On the other hand, the TMA avoids craniotomy and temporal lobe retraction and remains the preferable approach for cases of medial and purely posterior dehiscence (3). A recent comparative study by Schwartz et al. comparing middle cranial fossa and transmastoid surgical techniques showed that both techniques offer symptom resolution with minimal risk (5). The round window reinforcement technique is used in some centers to counteract the third window effect. In the literature, studies of this approach have shown poor long-term results in comparison with the high success rate of dehiscence plugging; thus, it is not advisable as the first-line intervention for SSCD (3, 10–14).

In addition, the removal of the thin bone fragments at the two limits of the dehiscence should not be a problem if it is performed by an experienced surgeon. It is important that this part of the process be completed superficially, with care to avoid penetration of the membranous labyrinth. This will facilitate and ensure the correct plugging, particularly in the case of a small dehiscence.

In our quaternary center, SSCD is repaired by plugging the canal through the middle cranial fossa approach. Intraoperatively, the most difficult step is the accurate identification of the dehiscent SSC over the superior surface of the temporal bone.

As we know, the arcuate eminence reflects the superior part of the SSC, but this is not always a clear landmark. The anatomy of the superior surface of the temporal bone is variable, and the landmarks are not always consistent, particularly if the petrous bone is highly pneumatized, in which case the arcuate eminence will not always be clearly visible. In these difficult cases, the neuronavigation technique proposed here will be of great assistance. By using this method, we directly situate the canal in the middle of our operative field. Making the craniotomy in exactly the right place is extremely valuable, especially since we make a small (3 cm × 3 cm) craniotomy, which means that it is especially important to make it in the optimal position.

For procedures carried out at our center between 2005 and 2010, this manual neuronavigation technique had not yet been developed; therefore, the craniotomy for SSC surgery was sometimes positioned more anterior or posterior, or more inferior or superior, to the exact position, making the procedure more challenging. In the face of these few particular cases, we developed this manual neuronavigation technique.

**Figure 5:**
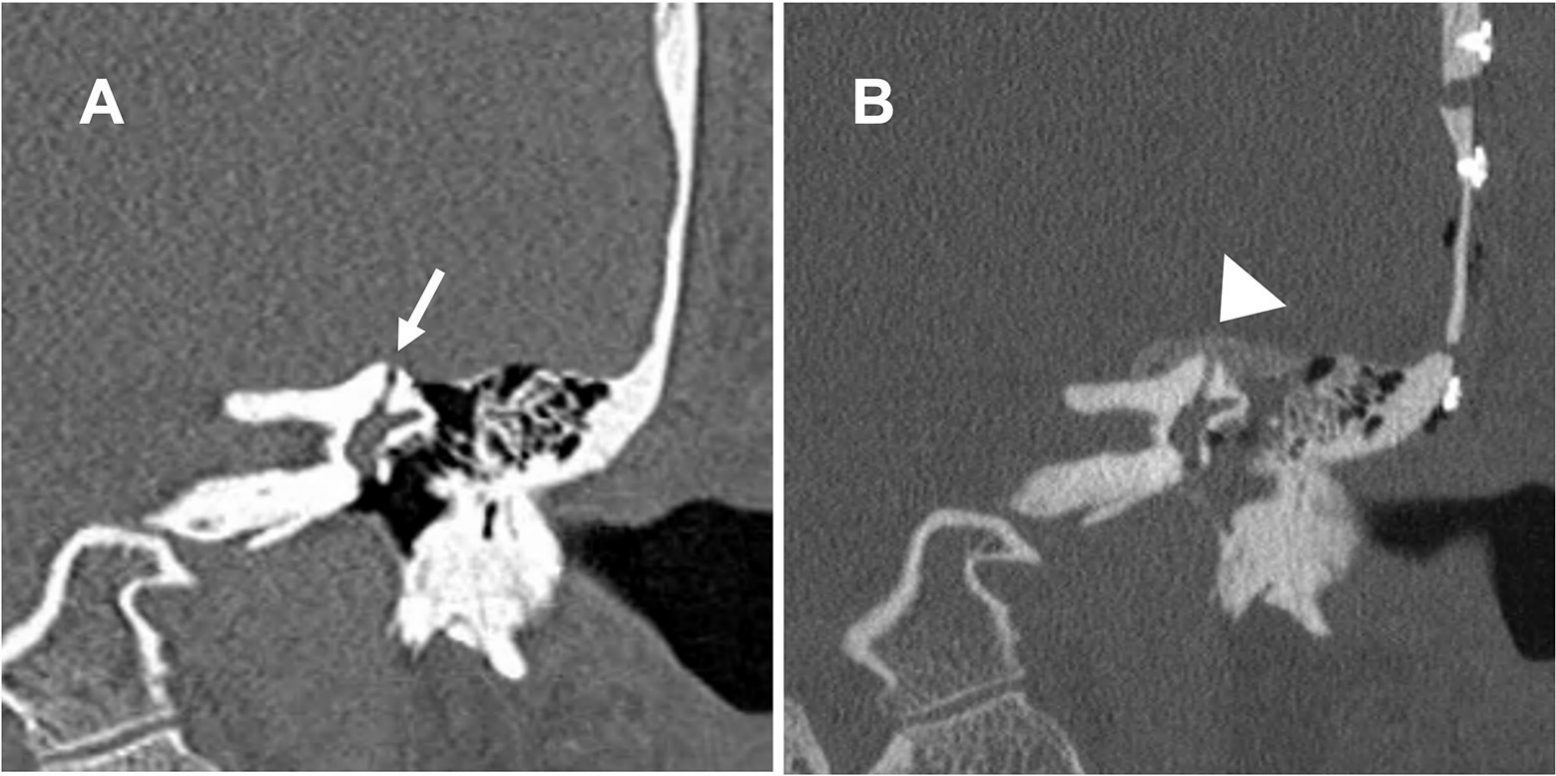
Coronal cut of a high-resolution CT scan of the left temporal bone. **(A)** The dehiscence of the left superior canal (arrow). **(B)** The bone dust plugging the dehiscence and covering the superior canal (arrowhead).

A neuronavigation system is a useful tool to precisely identify SSC dehiscence. However, there are several limitations regarding the cost of such systems that prevent some centers from providing them; additional limitations are the time consumed in installation and difficulties in using the neuronavigation system when the surgeon's experience is limited. Our study proposes a simple technique that can help otologic surgeons to identify the dehiscence of the SSC precisely and efficiently. The use of line A on the HRCT axial cut, which measures the distance from the dehiscence to the lateral cortical part of the supra-auricular squamous bone, provides a precise distance that can be measured during surgery. In our institution, we perform an HRCT on day 1 postoperatively to rule out any intracranial complications such as bleeding or hematoma. In addition to this, the postoperative HRCT scan confirms the correct identification and good plugging of the SSC dehiscence (Figure 5). Another prospective study to support and validate the proposed technique is planned.

## Conclusion

Although neuronavigation facilitates the surgical approach, we propose the use of surgical landmarks on a high-resolution CT scan as an alternative method to the use of a neuronavigation system. The proposed manual neuronavigation technique seems to be an accurate, safe, and cost-effective alternative technique for use in SSC dehiscence surgery. A randomized controlled trial is needed to confirm the accuracy of the proposed technique.

## Data availability statement

The original contributions presented in the study are included in the article/Supplementary material, further inquiries can be directed to the corresponding author.

## Ethics statement

Ethical review and approval was not required for the study on human participants in accordance with the local legislation and institutional requirements. Written informed consent for participation was not required for this study in accordance with the national legislation and the institutional requirements.

## Author contributions

All authors listed have made a substantial, direct, and intellectual contribution to the work and approved it for publication.
